# An analysis of the burden of colorectal cancer caused by high body mass index in 204 countries and regions worldwide from 1990 to 2023

**DOI:** 10.7189/jogh.16.04043

**Published:** 2026-02-27

**Authors:** Zeyu Wu, Yuncan Xing, Fangze Wei, Shiwen Mei, Qian Liu

**Affiliations:** 1 Department of Colorectal Surgery, Chinese Academy of Medical Sciences and Peking Union Medical College, National Cancer Center/National Clinical Research Center for Cancer/Cancer Hospital, Beijing, China; 2 State Key Laboratory of Molecular Oncology, Department of Clinical Laboratory, Chinese Academy of Medical Sciences and Peking Union Medical College, National Cancer Center/National Clinical Research Center for Cancer/Cancer Hospital, Beijing, China

## Abstract

**Background:**

Colorectal cancer (CRC) remains a leading global malignancy with a rising obesity-attributable burden. Emerging evidence highlights concerning trends in early-onset CRC and marked regional disparities, underscoring the need for comprehensive epidemiological assessments to inform targeted prevention strategies.

**Methods:**

Using Global Burden of Disease 2023 data, we analysed high body mass index (BMI)-related CRC deaths and disability-adjusted life years (DALYs) among adults (>40 years) from 1990–2023. We analysed both absolute counts and age-standardised rates, stratifying by sex, age, region, and sociodemographic index (SDI) categories. Decomposition analysis quantified the contributions of ageing, population growth, and epidemiological factors. We used Bayesian age-period-cohort analysis to project future trends.

**Results:**

From 1990 to 2023, the global number of high BMI-related CRC deaths increased more than 2-fold, accompanied by a corresponding marked increase in DALYs. Western Europe had the highest burden, while South Asia had the most rapid growth in deaths, as measured by the estimated annual percentage change. Generally, as SDI decreased, the corresponding numbers of deaths and DALYs decreased. Cluster analysis based on the estimated annual percentage changes in age-standardised rates of high BMI-related CRC deaths and DALYs identified distinct regional patterns, with significant decreases in these rates in Western Europe and high-income North America, contrasted by significant increases in South Asia and Central Sub-Saharan Africa. Decomposition analysis indicated that population growth was the primary driver of the rise in mortality, followed by population ageing, and these were partially offset by improvements in epidemiological risk. Projections suggest a continuing increase in the age-standardised death rates for both males and females by 2038.

**Conclusions:**

High BMI has become a key driver of CRC mortality and incidence worldwide. Reducing this burden requires efforts in healthy lifestyles, policy reforms, and international scientific cooperation.

In 2022, colorectal cancer (CRC) was responsible for an estimated 1.9 million new cases and 904 000 deaths globally [1]. Despite advances in early screening and diagnosis, which enabled timely intervention for a substantial proportion of patients, CRC remains a pressing public health issue as the third most diagnosed cancer type and the second leading cause of cancer mortality worldwide [2]. The development and progression of CRC are influenced by a range of risk factors. These include structural drivers such as population ageing and dietary patterns in high-income countries, as well as modifiable factors – notably elevated body mass index (BMI) and physical inactivity – which have been consistently associated with an increased risk of CRC [3]. Previous studies have reported some modifiable risk factors for CRC, with obesity being consistently identified as a major independent driver. This is largely because excess body fat promotes a state of chronic inflammation and hormonal imbalances, which can directly fuel cancer development in the colon, even in the absence of other risk factors [4–7]. Clinically, a high BMI can affect multiple carcinogenic pathways and is associated with adverse clinical outcomes and an overall poorer prognosis in CRC patients [8–12].

Although CRC typically occurs after the age of 50, emerging data indicate a concerning rise in early-onset cases [13]. In response, several countries have revised screening guidelines, lowering the recommended initiation age to 45 for average-risk individuals to optimise the balance between life-years gained and colonoscopy utilisation [14,15]. Notably, several studies have suggested that initiating CRC screening at age 40, rather than the current standard of age 50, is cost-effective, increases quality-adjusted life years, and further reduces CRC mortality and incidence [16–19]. Moreover, a recent study found that individuals aged 40–49 bear a substantial burden of CRC [20].

However, previous studies have typically focused on CRC burden of high BMI across all age groups and have rarely targeted the special high-risk population of those aged ≥40 years [21–23]. The newly released Global Burden of Disease (GBD) 2023 data set offers an opportunity to reassess the CRC burden, specifically in high-BMI populations of this group. Therefore, we aimed to quantify the global burden of CRC attributable to high BMI in adults aged >40 years from 1990 to 2023, and to project future trends. Our findings could provide comprehensive epidemiological evidence to inform targeted prevention and screening strategies for both classic and younger at-risk populations.

## METHODS

### Data source

We used data from the GBD 2023 database, a rigorously curated epidemiological repository providing systematic estimates of disease burden attributable to various risk factors (including high BMI) across global, regional, and national populations. The data set encompasses comprehensive metrics on incidence, prevalence, mortality, and disability-adjusted life years (DALYs) spanning 204 countries and territories from 1990 to 2023. We used the Global Health Data Exchange query tool for data extraction, where we specified ‘High Body Mass Index’ as the risk factor and ‘Colorectal Cancer’ as the associated cause. From this query, we obtained absolute numbers and age-standardised death rates (ASDRs) and DALYs attributable to high BMI in CRC among individuals aged >40 years. According to GBD 2023 data, a high BMI in adults is defined as >25 kg/m^2^.

### Outcomes

The primary outcomes were deaths, DALYs, and their ASDRs for CRC burden attributable to high BMI by age, sex, geographic region, and sociodemographic index (SDI) category, which represent the core epidemiological measures of risk. Secondary outcomes included temporal trends in the primary outcomes (quantified using the estimated annual percentage changes (EAPCs)), decomposition analyses of changes in death and DALY counts into contributions from population growth, ageing, and changes in epidemiological rates, and projected future trends.

### Statistical methods

We employed the SDI, a composite development metric strongly predictive of health outcomes. In addition, to quantify the drivers of high BMI-attributable CRC mortality and disability, we performed a decomposition analysis. This standardised approach, commonly applied in disease burden studies, isolates the distinct contributions of demographic and epidemiological forces. We generated age- and sex-stratified estimates to elucidate potential disparities in CRC burden trajectories.

#### Decomposition analysis

The decomposition analysis was based on the standard demographic decomposition framework developed by Das Gupta [24]. This method is specifically designed to decompose the temporal changes in ratios or counts into cumulative components (*e.g.* population growth, age structure, and changes in incidence rates), and it eliminates the base-year bias by taking the symmetric average of the weights at the two time points so provides critical insights into the underlying drivers of the changing burden of CRC attributable to high BMI.

#### Predictive analysis

To project the future burden of CRC attributable to high BMI, we employed the Bayesian age-period-cohort model. This model simultaneously evaluates temporal trends through age-, period-, and birth-cohort-specific effects while incorporating known demographic transitions. This framework is particularly suited for handling the complexity and uncertainty of epidemiological data, enabling robust long-term projections by explicitly accounting for the dynamic interactions between ageing and population growth over time. Additionally, we conducted a sensitivity analysis using exponential smoothing models to evaluate the robustness of the predictions.

### Statistics

All epidemiological metrics in the GBD database were reported with 95% uncertainty intervals (UIs). These intervals represent the total uncertainty generated through the entire analytical cascade, from primary data heterogeneity to model-based imputation. It was constructed using a Monte Carlo statistical framework comprising 1000 iterative simulations [23,25]. We summarised the long-term trend of the ASDR by computing the EAPC. This metric was derived by applying a linear regression to the natural logarithm of the ASDR over time. We computed the EAPC (Equation S1 in the **Online Supplementary Document**), where β is the model's slope coefficient, serving as a metric for the average annual percentage change. We determined the associated 95% confidence interval (CI) from the standard error of the slope estimate.

We used *R*, version 4.5.1 (R Core Team, Vienna, Austria) for all analyses.

### Ethical considerations

As this study employed de-identified, aggregate data from the publicly accessible repository, neither institutional review board approval nor individual informed consent was required. We adhered to relevant ethical guidelines and regulatory standards governing the utilisation of public health data for all methodological procedures. We conducted this study in accordance with the STROBE guidelines (Checklist S1 in the **Online Supplementary Document**) [26].

## RESULTS

### Global burden of CRC attributable to high BMI in adults >40 years

At the global level, the number of death cases increased from 42 286 (95% UI = 20 419, 66 614) in 1990 to 99 831 (95% UI = 50 719, 15 1523) in 2023, and the number of DALYs cases increased from 1 008 309 (95% UI = 488 414, 1 578 447) in 1990 to 2 349 559 (95% UI = 1 194 851, 3 534 099) in 2023 (**Table 1**; Figure S1 in the **Online Supplementary Document**). However, the estimated ASDRs of CRC attributable to high BMI were 3.41 (95% UI = 1.65, 5.39) in 1990 and 3.27 (95% UI = 1.66, 4.97) in 2023. The DALYs were 76.05 (95% UI = 36.83, 119.28) in 1990 and 76.24 (95% UI = 38.78, 114.75) in 2023 per 100 000 population. Temporal trend analysis revealed a reduction in the DALYs (EAPC = −0.22; 95% CI = −0.29, −0.15) and ASDRs (EAPC = −0.33; 95% CI = −0.39, −0.26) for CRC related to high BMI (Figure S2, Panels A and B in the **Online Supplementary Document**).

**Table 1:** Mortality cases, ASDRs, and DALYs of CRC attributed to high BMI for adults >40 years, 1990–2023

	1990		2023		1990–2023	1990		2023		1990-2023
	**n (95% UI)**	**ASDR (95% UI)**	**n (95% UI)**	**ASDR (95% UI)**	**EAPC (95% CI)**	**n (95% UI)**	**DALYs (95% UI)**	**n (95% UI)**	**DALYs (95% UI**	**EAPC (95% CI)**
**Global**	42 286 (20 419, 66 614)	3.41 (1.65, 5.39)	99 831 (50 719, 151 523)	3.27 (1.66, 4.97)	−0.33 (−0.39, −0.26)	1 008 309 (488 415, 1 578 447)	76.05 (36.83, 119.28)	2 349 599 (1 194 852, 3 534 099)	76.24 (38.78, 114.75)	−0.22 (−0.29, −0.15)
**SDI**										
High	35 237.17 (17 048.02, 55 603.24)	5.15 (2.49, 8.14)	69 725.62 (35 624.36, 105 249.94)	4.72 (2.41, 7.11)	−0.49 (−0.6, −0.38)	822 367.27 (398 859.71, 1 288 136.76)	117.75 (57.08, 184.66)	1 544 669.63 (789 109.56, 2 313 731.04)	110.05 (56.24, 164.54)	−0.47 (−0.57, −0.37)
High-middle	2999.59 (1407.52, 4860.79)	1.3 (0.61, 2.11)	14 035.95 (6943.6, 21 661.75)	2.05 (1.01, 3.16)	1.32 (1.28, 1.37)	84 993.34 (39 947, 137 504.25)	33.52 (15.76, 54.3)	366 847.53 (182 156.5, 560 655.78)	52.48 (26.05, 80.3)	1.27 (1.22, 1.32)
Middle	589.55 (271.66, 967.74)	0.46 (0.21, 0.76)	3987.82 (2006.99, 6691.94)	1.27 (0.64, 2.13)	3.24 (3.12, 3.36)	17 079.9 (7888.52, 28 192.96)	12.04 (5.55, 19.83)	113 266.91 (57 518.82, 189 001.69)	33.63 (17.04, 56.2)	3.31 (3.2, 3.42)
Low-middle	394.51 (165.91, 706.51)	0.31 (0.13, 0.56)	3001.93 (1457.08, 5447.92)	0.93 (0.45, 1.7)	3.26 (3.09, 3.44)	11 892.63 (5029.42, 21 185.66)	8.58 (3.61, 15.34)	91 204.06 (44 784.23, 163 603.88)	25.97 (12.7, 46.8)	3.34 (3.14, 3.53)
Low	862.77 (409.09, 1377.66)	0.78 (0.37, 1.25)	6098.49 (3155.48, 9683.27)	2.02 (1.04, 3.22)	2.99 (2.94, 3.05)	25 584.78 (12 167.95, 40 805.57)	21.04 (9.98, 33.62)	175 357.15 (90 870.55, 278 938.63)	54.22 (28.07, 86.3)	2.94 (2.9, 2.99)
**Europe**										
Central	3282.55 (1578.73, 5242.07)	6.56 (3.15, 10.5)	6536.41 (3368.4, 9736.71)	8.3 (4.28, 12.36)	0.68 (0.53, 0.83)	77 868.88 (37 509.56, 124 161.31)	152.26 (73.26, 243.08)	139 011 (71 756.89, 205 368.73)	185.84 (96, 274.29)	0.56 (0.41, 0.72)
Western	14 096.92 (6782.77, 22 550.32)	6.99 (3.37, 11.17)	20 755.76 (10 714.66, 31 593.5)	5.91 (3.05, 8.94)	−0.83 (−0.95, −0.71)	296 145.12 (142 784.2, 469 923.02)	154.28 (74.38, 244.28)	398 126.19 (206 684.78, 596 816.19)	129 (66.92, 192.5)	−0.88 (−1.01, −0.76)
Eastern	5204.58 (2525.31, 7977.56)	5.49 (2.66, 8.43)	8529.25 (4416.34, 12 789.73)	6.75 (3.5, 10.14)	0.56 (0.38, 0.74)	131 324.13 (64 077.56, 200 507.47)	136.7 (66.67, 209.02)	197 373.23 (102 546.65, 294 342.69)	159.35 (82.9, 237.74)	0.3 (0.12, 0.47)
**America**										
Andean Latin	127.76 (60.37, 213.5)	1.94 (0.92, 3.25)	698.96 (358.33, 1088.62)	3.26 (1.67, 5.09)	1.6 (1.4, 1.8)	3285.62 (1560.77, 5477.05)	46.9 (22.25, 78.24)	16 868.5 (8671.4, 25 987.67)	76.71 (39.41, 118.33)	1.52 (1.31, 1.74)
Southern Latin	1063.3 (513.03, 1690.58)	7.03 (3.38-11.21)	2777.04 (1467.28, 4202.28)	8.62 (4.56, 13.01)	0.69 (0.6, 0.78)	24 617.49 (11 958.98, 38 781.42)	157.76 (76.57, 248.99)	60 287.49 (31 919.04, 90 006.39)	195 (103.3, 290.67)	0.75 (0.67, 0.82)
High-income North	7934.83 (4026.03, 12 044.13)	6.65 (3.38, 10.09)	12 461.82 (6469.84, 18 527.01)	5.57 (2.89, 8.26)	−0.71 (−0.82, −0.6)	179 357.4 (91 669.19, 270 314.67)	157.15 (80.33, 236.73)	291 031.99 (150 884.26, 429 741.15)	140.89 (72.92, 207.77)	−0.54 (−0.63, −0.44)
Central Latin	519.92 (246.81, 832.18)	1.95 (0.93, 3.13)	3230.37 (1664.47, 4750.27)	3.6 (1.86, 5.31)	1.98 (1.86, 2.09)	13 516.87 (6441.74, 21 581.58)	47.05 (22.4, 75.25)	82 681.54 (42 718.97, 120 757.89)	89.58 (46.25, 131.01)	2.11 (1.99, 2.23)
Tropical Latin	559.31 (265.06, 906.19)	1.94 (0.92, 3.15)	3744.85 (1862.16, 5740)	4.14 (2.06, 6.35)	2.52 (2.38, 2.65)	14 498.49 (6879.43, 23 449.05)	45.93 (21.76, 74.36)	92 514.5 (46 203.38, 140 879.63)	100.62 (50.25, 153.25)	2.56 (2.44, 2.69)
**Asia**										
Central	490.75 (233.68, 787.44)	3.23 (1.53, 5.21)	826.48 (425.03, 1280.86)	2.82 (1.44, 4.4)	0 (−0.15, 0.16)	13 690.67 (6548.95, 21 840.18)	85.55 (40.83, 137.06)	22 145 (11 491.81, 34 011.63)	69.9 (36.14, 107.79)	−0.32 (−0.45, −0.2)
East	4052.05 (1685.49, 6990.04)	1.32 (0.54, 2.29)	15 636.49 (7280.64, 25 842.48)	1.95 (0.91, 3.22)	0.71 (0.55, 0.86)	120 772.62 (51 077.93, 206 950.29)	37.33 (15.68, 64.22)	407 461.9 (191 800.08, 661 745.3)	51.01 (24.04, 82.79)	0.42 (0.26, 0.58)
High-income Pacific	1543.87 (713.51, 2548.86)	2.31 (1.06, 3.83)	4865.51 (2251.25, 8437.4)	2.82 (1.34, 4.73)	0.33 (0.26, 0.4)	39 463.32 (18 384.67, 64 331.57)	57.08 (26.53, 93.24)	94 802.24 (45 080.37, 159 414.58)	65.96 (32.03, 108.08)	0.16 (0.09, 0.23)
South	292.1 (117.58, 545)	0.14 (0.06, 0.27)	3687.41 (1758.26, 6244.27)	0.69 (0.33, 1.18)	5.17 (4.96, 5.38)	9195.64 (3720.74, 17 023.85)	4.15 (1.67, 7.72)	110 000.76 (53 238.06, 184 516.33)	19.51 (9.41, 32.81)	5.06 (4.85, 5.26)
Southeast	474.26 (210.2, 837.32)	0.54 (0.24, 0.97)	4194.2 (2078.27, 6955.77)	1.68 (0.83, 2.8)	3.52 (3.4, 3.65)	14 421.27 (6468.34, 25 145.88)	15.17 (6.75, 26.64)	127 253.91 (63 606.26, 209 166.14)	48.33 (24.09, 79.68)	3.63 (3.52, 3.75)
**Africa**										
Caribbean	254.29 (123.03, 400.57)	2.89 (1.4, 4.55)	883.45 (444.59, 1414.94)	4.68 (2.35, 7.49)	1.56 (1.48, 1.65)	6343.21 (3075.43, 10 010.59)	71.24 (34.54, 112.42)	20 890.61 (10 587.31, 33 028.12)	110.52 (56.04, 174.77)	1.45 (1.35, 1.56)
Central Sub-Saharan	30.34 (12.21, 56.53)	0.38 (0.15, 0.71)	290.49 (124.54, 524.84)	1.31 (0.56, 2.39)	3.85 (3.57, 4.13)	956.4 (391.18, 1776.67)	10.84 (4.39, 20.24)	9327.53 (40 24.27, 16 754.22)	37.63 (16.13, 67.84)	3.89 (3.58, 4.2)
Eastern Sub-Saharan	95.02 (38.07, 177.97)	0.35 (0.14, 0.65)	731.96 (347.24, 1351)	1.04 (0.49, 1.95)	3.41 (3.21, 3.61)	3 005.62 (1216.71, 5571.57)	10.12 (4.07, 18.86)	22 495.23 (10 800.95, 40 655.54)	29.43 (14.09, 53.73)	3.3 (3.08, 3.51)
Southern Sub-Saharan	199.35 (93.91, 336.75)	2.3 (1.08, 3.9)	655.74 (311.11, 1050.85)	3.02 (1.43, 4.86)	0.77 (0.47, 1.06)	5091.9 (2391.55, 8549.10)	54.3 (25.56, 91.36)	17 260.77 (8124.59, 27 537.65)	73.82 (34.89, 117.89)	0.94 (0.62, 1.26)
Western Sub-Saharan	205.33 (92.45, 360.95)	0.68 (0.31, 1.21)	1175.82 (576.42, 2125.97)	1.61 (0.79, 2.93)	2.52 (2.38, 2.66)	6245.95 (2835.02, 10 958.06)	19.29 (8.72, 33.89)	35 337.67 (17 363.21, 63 497.61)	43.39 (21.3, 78.16)	2.35 (2.2, 2.51)
**Oceania**										
Australasia	626.04 (314.2, 969.64)	7.95 (3.98, 12.36)	1099.68 (565.22, 1683.54)	5.43 (2.81, 8.28)	−1.39 (−1.49, −1.28)	14 503.36 (7294.85, 22 197.04)	186.06 (93.46, 285.17)	22 433.7 (11 724.01, 33 893.77)	122.92 (64.38, 185.16)	−1.52 (−1.63, −1.42)
Oceania	12.57 (5.88, 21.32)	1.39 (0.65, 2.35)	56.56 (27.61, 98.39)	2.16 (1.04-3.77)	1.17 (1.09, 1.25)	369.22 (173.28, 627.59)	34.63 (16.27, 58.82)	1670.51 (819.52, 2903.16)	54.81 (26.75, 95.42)	1.22 (1.15, 1.28)

ASDR – age-standardised death rate, BMI – body mass index, CI – confidence interval, CRC – colorectal cancer, DALY – disability-adjusted life-year, EAPC – estimated annual percentage change, SDI – sociodemographic index, UI – uncertainty interval

### Regional burden of CRC attributable to high BMI in adults >40 years

**Figure 1:**
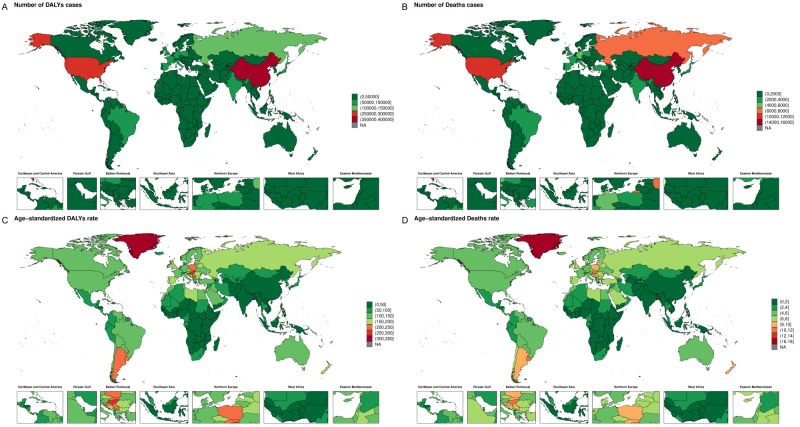
DALYs, deaths, age-standardised DALYs, and ASDRs of CRC that are attributed to high BMI for adults aged >40 years in 2023. **Panel A.** Number of DALY cases. **Panel B.** Number of death cases. **Panel C.** Age-standardised DALY rate. **Panel D.** Age-standardised death rate. ASDRs – age-standardised rates, BMI – body mass index, CRC – colorectal cancer, DALYs – disability-adjusted life years, NA – not available.

**Figure 3:**
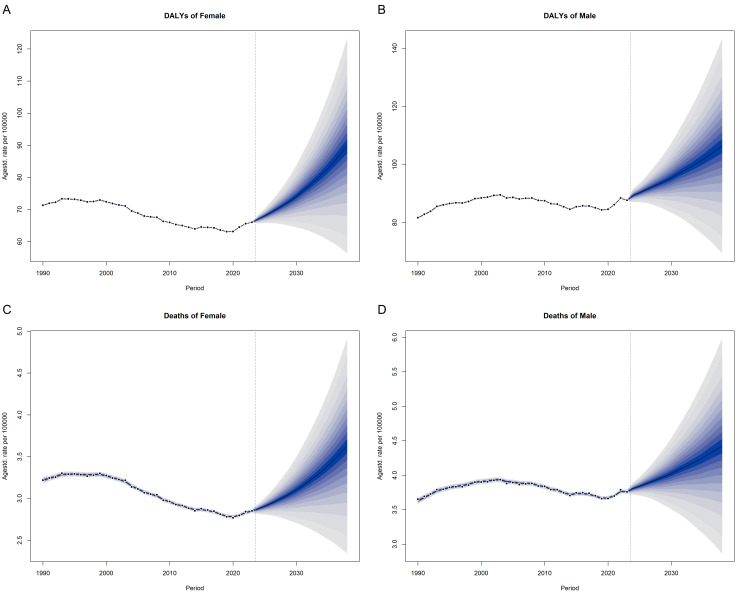
Temporal trends in the observed and predicted global disease burden of CRC attributed to high BMI among adults aged >40 years, as estimated by the BAPC model from 1990 to 2038. **Panel A.** DALYs of female. **Panel B.** DALYs of male. **Panel C.** Deaths of female. **Panel D.** Deaths of male. BAPC – Bayesian age-period-cohort analysis, BMI – body mass index, CRC – colorectal cancer, DALY – disability-adjusted life year.

In 2023, Western Europe reported one of the highest DALY counts of 398 126 (95% UI = 206 685, 596 816) (**Figure 3**, Panel A; Figure S1, Panel A in the **Online Supplementary Document**). Western Europe also had the highest absolute mortality burden of CRC attributable to high BMI, with 20 756 (95% UI = 10 715, 31 594) deaths, accounting for 21% of global deaths (**Figure 1**, Panel B; Figure S3, Panel A in the **Online Supplementary Document**). These numbers were accompanied by a DALY of 129 (95% UI = 66.92, 192.50) and an ASDR of 5.91 (95% UI = 3.05, 8.94) (**Figure 1**, Panels A–D; Figure S3, Panel B in the **Online Supplementary Document**), both of which showed negative EAPCs of −0.83 (95% CI = −0.95, −0.71) and −0.88 (95% CI = −1.01, −0.76), respectively (Figure S2 in the **Online Supplementary Document**). Additionally, East Asia exhibited the second-highest disease burden, with 15 636 deaths (95% UI = 7281, 25 842) and 1.95 ASDR (95% UI = 0.91, 3.22), while demonstrating an increasing trend (EAPC = 0.71; 95% UI = 0.55, 0.86) (Figure S3 in the **Online Supplementary Document**). In 2023, the burden of CRC mortality was also considerable in Central (DALY = 139 011; 95% UI = 71 757, 205 369) and Eastern Europe (DALY = 197 373; 95% UI = 102 547, 294 343). The Americas have also demonstrated marked disparities in CRC mortality and DALYs across regions. High-income North America, including Canada and the USA, had the third highest disease burden, reporting 12 461.82 (95% UI = 6469.84, 18 527.01) deaths, with an ASDR of 5.57 (95% UI = 2.89, 8.26) and a DALY of 291 032 (95% UI = 150 884, 429 741). Similar to Western Europe, the trend in North America showed a slight decline (EAPC of DALYs = −0.54; 95% UI = −0.63, −0.44). Notably, South Asia had the lowest ASDR (0.69; 95% UI = 0.33, 1.18) and DALY (19.51; 95% UI = 9.41, 23.81) rates. In contrast, South Asia also showed an increasing trend over time, with EAPC values of 5.17 (95% CI = 4.96, 5.38) for ASDR and 5.06 (95% CI = 4.85, 5.26) for DALYs.

In 2023, among SDI regions, the highest number of CRC deaths attributable to high BMI was observed in high SDI regions (n = 69 725; 95% UI = 35 624, 105 250), and this pattern was also observed for ASDRs (Figure S4, Panels A and B in the **Online Supplementary Document**). Generally, as SDI decreased, the corresponding number of deaths decreased, with similar results observed in DALYs.

Temporal trend analysis revealed increases in deaths and DALYs across all SDI regions (Figure S5, Panels A and B in the **Online Supplementary Document**), with the growth in high SDI being the sharpest. In contrast, the ASDRs in high SDI increased from 1990 to 2000, then decreased until 2020, and remained steady from 2020 to 2023, while other SDI regions showed a slow but steady increase from 1990 to 2023.

In the GBD regions, the CRC disease burden attributable to high BMI varied significantly. To identify regions with similar changes in disease burden, we conducted stratified cluster analyses. Among the regions, high-income North America, Central Asia, Western Europe and Australasia exhibited a significant decreasing trend, while four regions (Southeast Asia, Central Sub-Saharan Africa, Eastern Sub-Saharan Africa, and South Asia) showed a significant increasing trend (**Table 1**; Figure S6 in the **Online Supplementary Document**). From the regional data between 1990 and 2023, South Asia had the most pronounced increases in ASDR (EAPC = 5.17; 95% CI = 4.96, 5.38) and age-standardised DALY rate (EAPC = 5.06; 95% CI = 4.85, 5.26).

### Temporal trends in CRC burden attributable to high BMI in adults aged >40 years, 1990–2023

The number of deaths increased steadily over time, with males consistently showing higher numbers and ASDRs than females (Figure S7 in the **Online Supplementary Document**). For the trend observed in DALYs, both the number and age-standardised rates have risen steadily. After 2000, the difference in the numbers and age-standardised rates between males and females has gradually increased. All the data indicated a grave burden due to CRC attributable to high BMI in adults >40 years over the three decades.

### Age- and sex-specific burden of CRC attributable to high BMI in adults aged >40 years, 2023

Global analyses showed substantial age- and sex-specific disparities in the high-BMI-attributable burden of CRC in 2023. Absolute mortality and DALYs showed an age-dependent escalation, peaking at 60–79 years (Figure S8, Panel A in the **Online Supplementary Document**). Males consistently had a greater burden magnitude than females, with the largest sex differences observed at 65–69 years (Figure S9, Panel A in the **Online Supplementary Document**). The ASDRs exhibited rapid growth after age 60, peaking around age 90 (Figure S8, Panel B, in the **Online Supplementary Document**). This geriatric acceleration was particularly pronounced in males, demonstrating the risk rise that underscores their disproportionate vulnerability in the elder group (Figure S9, Panel B in the **Online Supplementary Document**).

### Decomposition analysis

**Figure 2:**
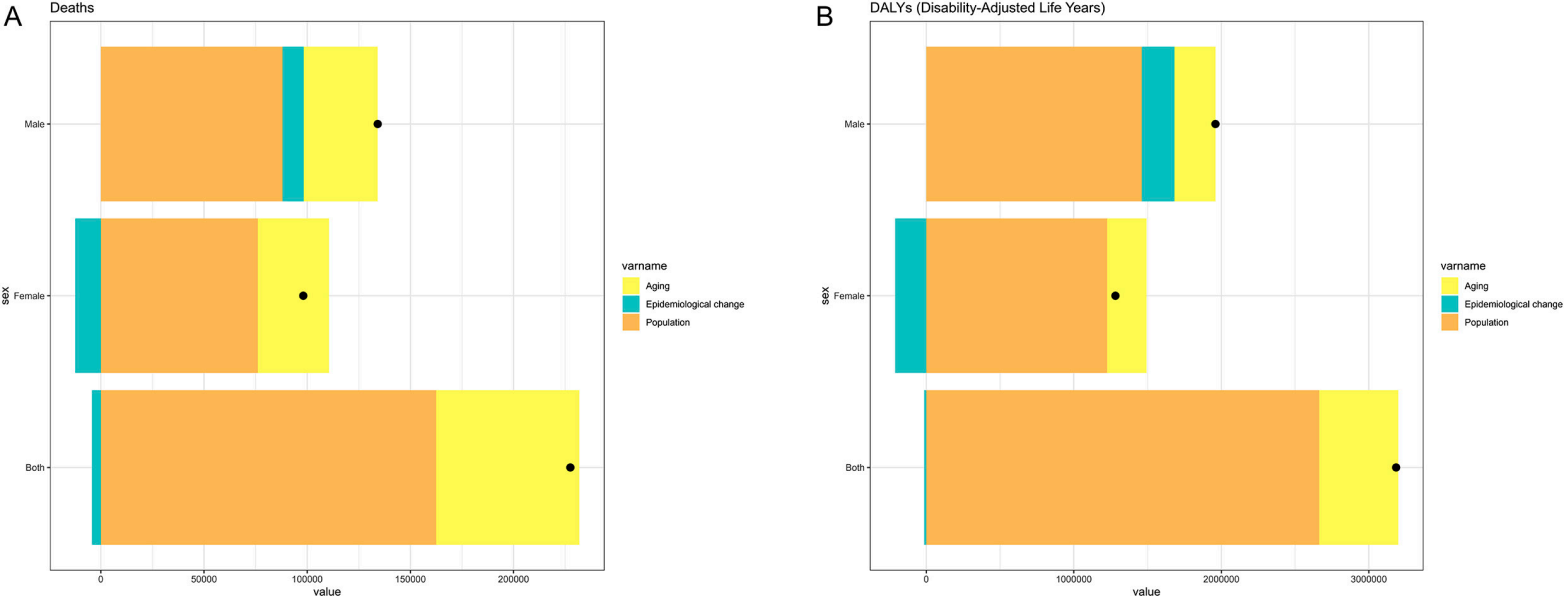
Decomposition analysis of ageing, population growth, and epidemiological change contributing to changes in deaths and DALYs for CRC attributable to high BMI for adults >40 years. **Panel A.** Deaths. **Panel B.** DALYs. BMI – body mass index, CRC – colorectal cancer, DALYs – disability-adjusted life years.

We found differential contributions of ageing, population growth, and epidemiological changes to the number of CRC deaths and DALYs attributable to high BMI in 2023 (**Figure 2**, Panels A and B). Regarding CRC mortality, ageing accounted for 69 392.97 (30.5%) deaths, population growth contributed 162 480.53 (71.42%) deaths, while epidemiological changes exerted a mitigating effect, with a reduction of 4358.55 (−1.92%) deaths. Among males, ageing contributed to 35 875.75 (26.74%), population growth to 88 075.27 (65.64%), and epidemiological changes to 10 226.44 (7.62%) deaths. Conversely, in females, ageing contributed to 34 410.31 (35.08%) deaths, and population growth to 76 112.63 (77.59%) deaths, while epidemiological changes reduced 12 420.94 (−12.66%) deaths. Regarding DALYs, ageing contributed 535 462.61 (16.81%), population growth contributed 2 663 579.18 (83.63%), and epidemiological changes reduced the number by −4358.55 (−0.45%). When stratified by sex, the numbers were 278 274.54 (14.2%) for ageing, 1 461 072.21 (74.53%) for population growth, and 220 902.93 (11.27%) for epidemiological changes in males, and 267 682.90 (20.88%) for ageing, 1 225 553.88 (95.59%) for population growth, and −211 144.83 (−16.47%) for epidemiological changes in females.

These findings underscore the substantial influence of ageing in elevating the risk of CRC-related mortality and DALYs attributable to high BMI in both sexes. While epidemiological shifts exerted a modest attenuating effect at the population level, demographic expansion remained a pivotal driver of rising death and DALY counts. Notably, despite the exacerbating roles of ageing and population growth in mortality trends, advancements in medical technology and the adoption of targeted health interventions have mitigated this upward trajectory. This moderating influence was particularly pronounced among females.

Collectively, the results indicate that while demographic and ageing-related factors amplify the burden of high-BMI-associated CRC, robust public health policies and ongoing medical innovation can partially counteract these adverse effects, thereby attenuating mortality and DALY risks.

### Predictions of sex-specific CRC burden attributable to high BMI in adults aged >40 years

The prediction of Bayesian age-period-cohort model showed that the disease burden of CRC would consistently increase (**Figure 3**, Panels A–D). In 2038, the predicted ASDRs was 3.644 (95% CI = 2.988, 4.300) for females and 4.444 (95% CI = 3.649, 5.239) for males per 100 000 population. For the DALYs, the values were 89.845 (95% CI = 72.825, 106.865) for females and 106.514 (95% CI = 87.661, 125.367) for males. The robustness of our predictions was further supported by the exponential smoothing model, which independently replicated the key finding of increasing CRC burden trends in both males and females (Table S1 in the **Online Supplementary Document**).

## DISCUSSION

We presented the first comprehensive assessment of the global burden of CRC attributable to high BMI in individuals aged >40 years across 204 countries and territories from 1990 to 2023. The global ASDRs and DALY rates remained relatively stable. However, it masked a substantial increase in absolute burden, with the absolute number of deaths and DALYs more than doubling. This divergence can be attributed to a combination of global population growth and ageing, coupled with a rising prevalence of obesity and the widespread adoption of modern lifestyles [27,28]. These findings underscore the escalating global burden of CRC linked to high BMI and highlight an urgent need for targeted public health policies to curb this growing threat. Addressing this reality demands a dual-track public health strategy: first, sustaining efforts to reduce the risk for the middle-aged and elderly population through prevention and early detection; and, more urgently, planning for the structural healthcare demands imposed by larger and older populations. The decline in EAPCs in the temporal trend analysis undoubtedly validates current achievements in global health and hygiene interventions. However, it is also necessary to conduct targeted, stratified research by region, country, gender, and age group to better understand the distribution of disease burden and to formulate detailed, targeted measures.

Regional heterogeneity in CRC burden showed significant inequities in obesity management and cancer control. Western Europe and North America sustained the highest absolute burdens but have achieved declining ASDRs. This improvement likely reflects sustained investments over the past decades, including wider access to colonoscopy, advances in multidisciplinary treatment models, and the implementation of obesity-related policies [29,30]. In contrast, emerging economies, particularly East Asia and South Asia, show alarming increases in CRC burden. These upward trends are closely associated with rapid economic development and urbanisation [31]. Improved healthcare infrastructure in these regions likely contributes to higher reported incidence through better diagnosis and increased healthcare-seeking behaviour. Concurrently, high rates of tobacco use and alcohol consumption persist [32,33], while urbanisation has led to significant reductions in physical activity due to sedentary jobs and reliance on motorised transport [34]. These external factors contribute to internal physiological disturbances, including impaired glucose metabolism, insulin resistance, gut microbiota dysbiosis, and chronic low-grade inflammation. This inflammatory environment triggers cytokine signalling that promotes colorectal carcinogenesis [35]. Therefore, comprehensive public health strategies – including stricter tobacco controls, public education on healthy eating, and programmes to encourage physical activity – are essential to reduce future CRC burden in these transitioning populations.

Cluster analysis highlighted distinct regional priorities for CRC control. In high-risk regions, such as South Asia and sub-Saharan Africa, there is a pronounced need for cost-effective screening strategies combined with foundational obesity prevention programmes, such as school-based nutrition initiatives. Stabilising regions (*e.g.* Western Europe/North America) must address rising youth obesity to avert future rebounds. Meanwhile, transitional economies (*e.g.* East Asia) require rapid scale-up of CRC registries and screening infrastructure.

Our findings indicated a steady increase in both the number of deaths and DALYs attributable to BMI in low, low-middle, and middle SDI regions between 1990 and 2023. This trend may be partly explained by modern lifestyle transitions, characterised by shifts in dietary patterns and reduced physical activity, which have contributed to rising obesity rates in these populations. Access to advanced medical facilities and high-quality healthcare systems is critical for improving CRC survival outcomes. A multicentre study of 15 958 patients demonstrated a significant disparity in 30-day postoperative mortality between resource-limited and high-income countries, with mortality being four times higher in the former (*P* < 0.05) [36]. In low- and middle- SDI regions, priorities must focus on foundational investments, including establishing and strengthening cancer registries, implementing cost-effective strategies for early detection and diagnosis, and improving overall access to healthcare services. In contrast, high-SDI regions, such as North America and Western Europe, showed a substantial absolute disease burden; however, their negative EAPC values indicated a favourable decline in CRC burden over the period. Public health efforts in these regions should focus on optimisation and equity, with emphasis on precise, group-level obesity prevention through community-based physical activity programmes, dietary interventions, and intensified health education campaigns.

Another key factor is biological and social vulnerability, particularly regarding gender and age [37]. Males accounted for >60% of the global CRC burden, with disparities widening after 2000. This aligns with evidence of higher visceral adiposity, greater exposure to alcohol and tobacco, androgen-driven tumorigenesis, and the absence of the oestrogen’s protective effects [38–40]. Age-specific analyses highlighted critical risk windows: absolute CRC burden peaked at ages 65–69, while rates per 100 000 are highest at ≥90, reflecting the cumulative impact of prolonged obesity exposure. The sharp increase in rates after age 60 suggests that geriatric populations, particularly males with lifelong elevated BMI, should be prioritised for screening. Social determinants, including delayed healthcare access in rural areas and gender norms that discourage preventive care among men, likely amplify these biological risks [41].

The decomposition model identified three main forces shaping CRC mortality. First, population growth was global but most pronounced in Africa and South Asia. Second, population ageing disproportionately affected high- and long-lived societies. Third, epidemiological changes reflected medical advances. Despite these factors, population growth and ageing remained dominant drivers, particularly in transitioning economies where healthcare infrastructure lags behind demographic change [42,43]. Addressing these challenges requires long-term investment in specialised healthcare infrastructure, including training oncological and surgical workforce, expanding endoscopic and radiotherapy capacity, and developing clinical guidelines tailored to the increasing cohort of older, multi-morbid patients. Epidemiological shifts, such as enhanced screening, optimised chemotherapy regimens, and public health policies, have exerted a protective influence on CRC outcomes. Notably, this buffer effect shows sexual dimorphism, being more pronounced in females. This may reflect gender-based differences in healthcare engagement, including higher screening adherence and greater utilisation of anti-obesity interventions, as well as biological factors such as oestrogen-mediated protective pathways and gut microbiota interactions [44–46].

Our model provided a plausible projection of the CRC incidence attributable to high BMI over the next ten years. This anticipated increase in CRC burden highlights the need for a proactive and nuanced policy response. However, these projections are conditional on current risk factor trends and do not account for the dynamic impact of future organised screening programmes, which may initially increase case detection before ultimately reducing late-stage disease. The model's accuracy is also limited by the historical data used as inputs. Accordingly, a two-pronged strategy is recommended. First, careful planning of phased, organised screening programmes is essential, with explicit consideration of cost-effectiveness and efforts to reduce rural-urban disparities. Second, primary prevention initiatives targeting modifiable risk factors, such as obesity, must be reinforced. Future modelling work that incorporates the potential effects of policy interventions and emerging treatments will be crucial to validate and dynamically update these public health forecasts.

Despite the comprehensive scope of this analysis, several inherent limitations should be acknowledged. First, the ecological nature of the data prevents inference at the individual level, meaning that observed population-level associations may not directly reflect individual risk. Second, our reliance on modelled estimates warrants caution. Estimates for some countries – particularly those with low-income, low-quality, or underdeveloped vital registration and cancer incidence data – were inherently uncertain, likely leading to underestimation of the burden of high BMI-attributable CRC in these regions. Additionally, the estimated burden may be influenced by residual confounding from factors not fully captured in our models, such as dietary patterns, educational attainment, social capital, or governance quality [47]. Nevertheless, the use of standardised GBD methodologies ensured robust cross-national comparability, providing valuable insights for cancer prevention and policy development. Future research should focus on high-quality, multidimensional studies to better inform the prevention and control of CRC.

## CONCLUSIONS

We found a substantial global increase in absolute deaths and DALYs from CRC attributable to high BMI between 1990 and 2023. However, ASDRs and DALYs showed a slight decline, suggesting that progress has partially offset the effects of demographic growth. The burden was the highest in Western Europe and other high-SDI regions, while alarmingly rising trends were evident in South Asia and East Asia. Decomposition analysis identified population growth and ageing as the main drivers of the increasing burden, partially mitigated by positive epidemiological trends, particularly among females. Significant sex and age disparities persisted, with males and older adults bearing the greatest burden. Projections indicated a continued rise in CRC attributable to high BMI, highlighting the urgent need for targeted interventions, especially in rapidly developing regions experiencing worsening trends.

## Additional material


Online Supplementary Document
