# Supplementary material for: An analysis of the burden of colorectal cancer caused by high body mass index in 204 countries and regions worldwide from 1990 to 2023
Source: J Glob Health. 2026 Feb 27;16:04043. doi: 10.7189/jogh.16.04043 (PMC12945346; doi:10.7189/jogh.16.04043)
Supplement: Online Supplementary Document [file jogh-16-04043-s001.pdf]

Supplement to: Wu Z, Xing Y, Wei F, Mei S, Liu Q. An analysis of the burden of colorectal cancer caused by high body mass index in 204 countries and regions worldwide from 1990 to 2023. J Glob Health. 2026;16:04043.

Figure S1. The global disease burden of CRC attribute to high BMI for adults over 40 years old from 1990 to 2023. **Panel A.** The numbers and ASDRs of deaths. **Panel B.** The numbers and ASDRs of DALYs.

ASDRs, age-standardized rates; BMI, body mass index; CRC, colorectal cancer; DALYs, disability-adjusted life years.

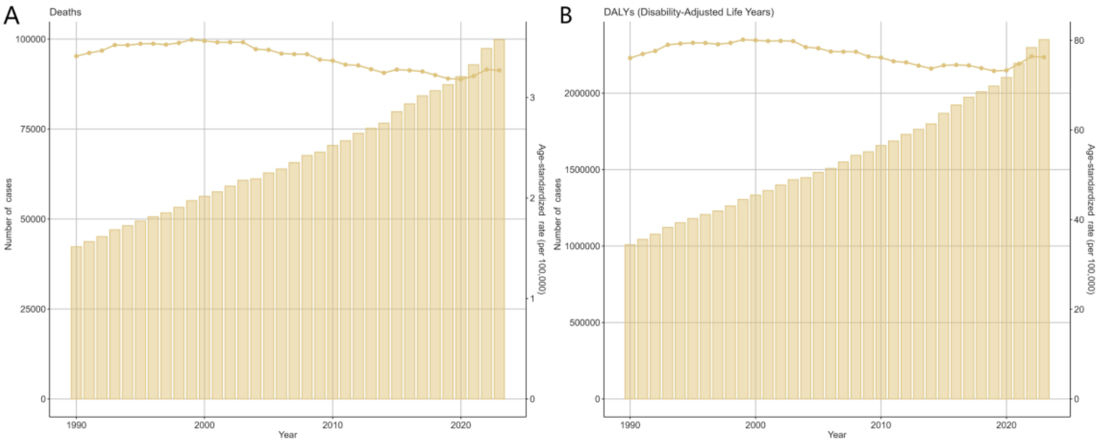

Figure S2. The EAPCs of the ASDRs of DALYs and ASDRs of deaths of CRC attribute to high BMI for adults over 40 years old globally. **Panel A.** The EAPCs of the ASDRs of DALYs. **Panel B.** The EAPCs of the ASDRs of deaths.

ASDRs, age-standardized rates; BMI, body mass index; CRC, colorectal cancer; DALYs, disability-adjusted life years; EAPC, estimated annual percentage change; NA, not available.

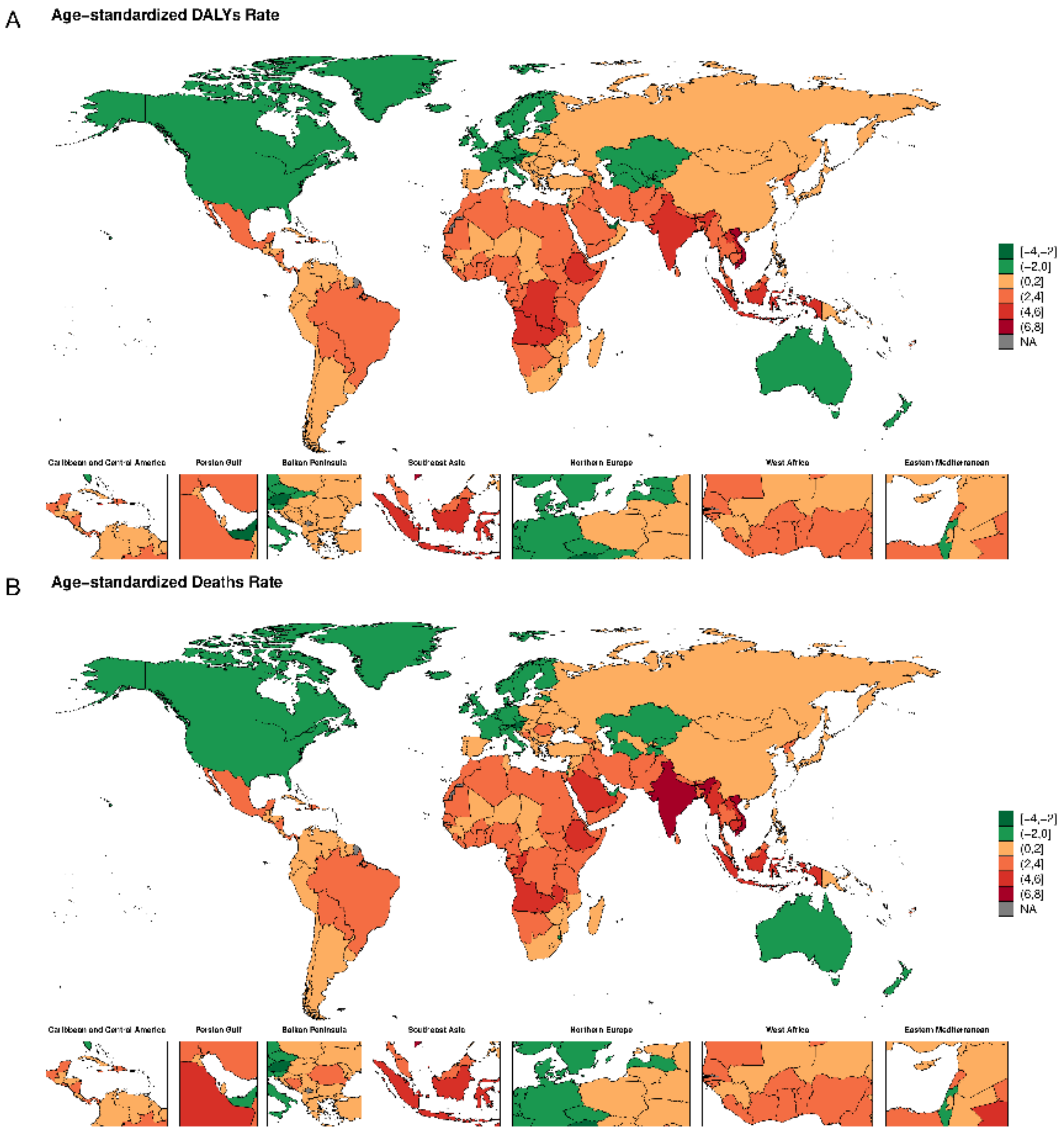

Figure S3. The disease burden of CRC in different GBD regions attribute to high BMI for adults over 40 years old in 2023. **Panel A.** The DALYs and deaths cases. **Panel B.** The ASDRs of DALYs and deaths.

ASDRs, age-standardized rates; BMI, body mass index; CRC, colorectal cancer; DALYs, disability-adjusted life years; GBD, the Global Burden of Disease database.

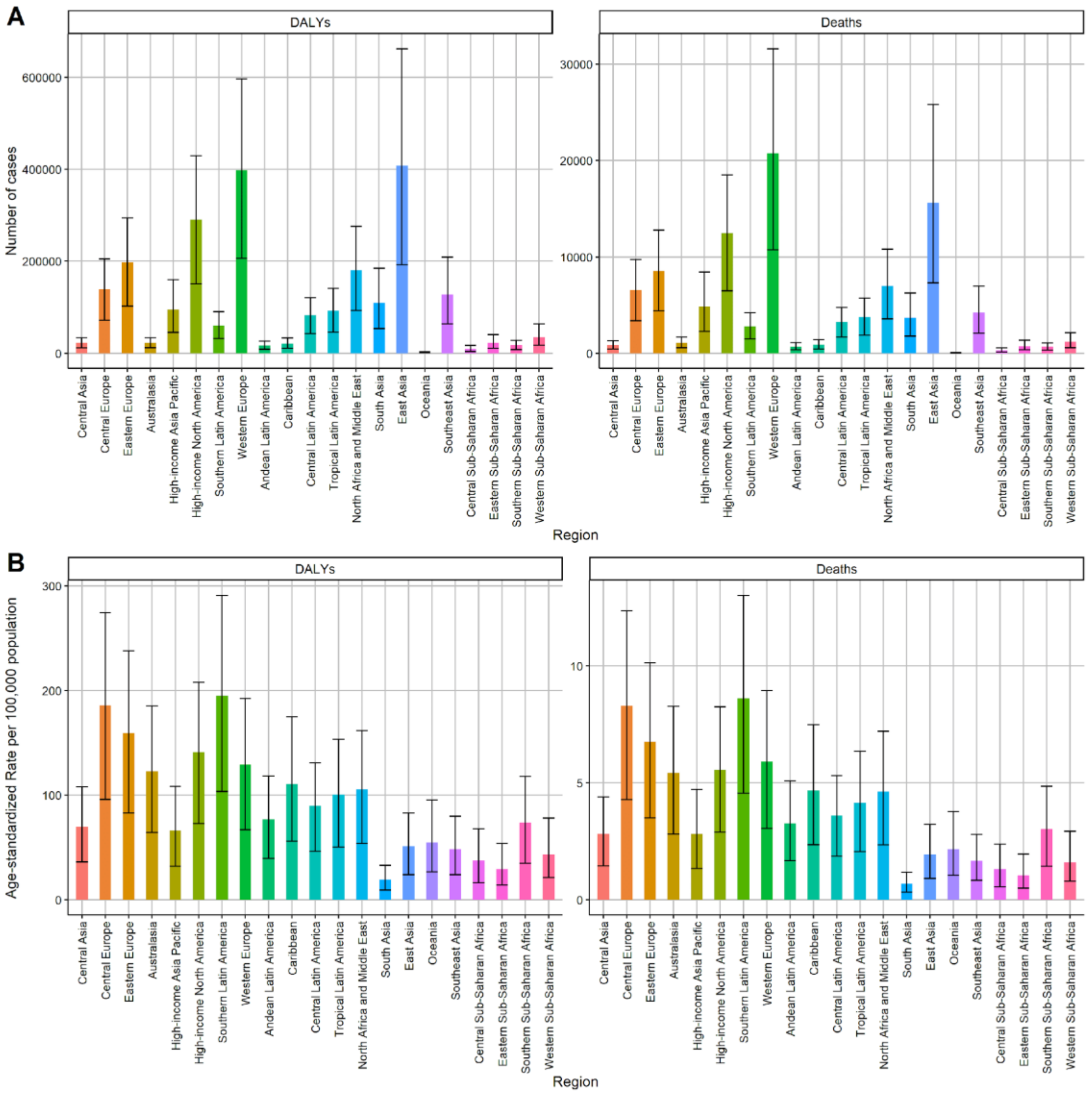

Figure S4. The disease burden of CRC in different SDI regions attribute to high BMI for adults over 40 years old in 2023. **Panel A.** The numbers of DALYs cases. **Panel B.** The numbers of deaths cases. **Panel C.** The ASDRs of DALYs. **Panel D.** The ASDRs of deaths.

ASDRs, age-standardized rates; BMI, body mass index; CRC, colorectal cancer; DALYs, disability-adjusted life years; SDI, socio-demographic index.

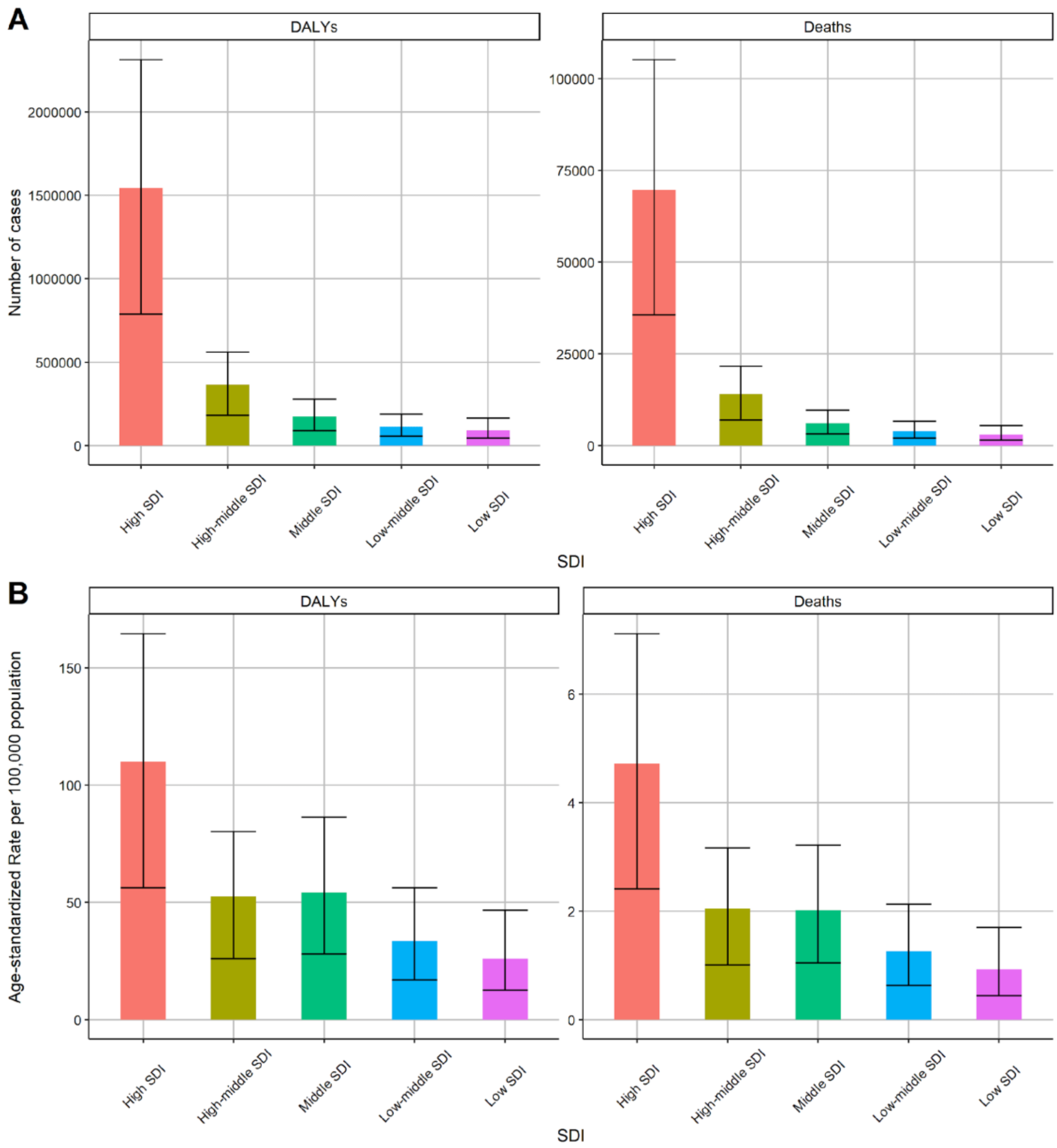

Figure S5. Temporal trends of the disease burden of CRC in different SDI regions attribute to high BMI for adults over 40 years old from 1990 to 2023. **Panel A.** The numbers of DALYs cases. **Panel B.** The numbers of deaths cases. **Panel C.** The ASDRs of DALYs. **Panel D.** The ASDRs of deaths.

ASDRs, age-standardized rates; BMI, body mass index; CRC, colorectal cancer; DALYs, disability-adjusted life years; SDI, socio-demographic index.

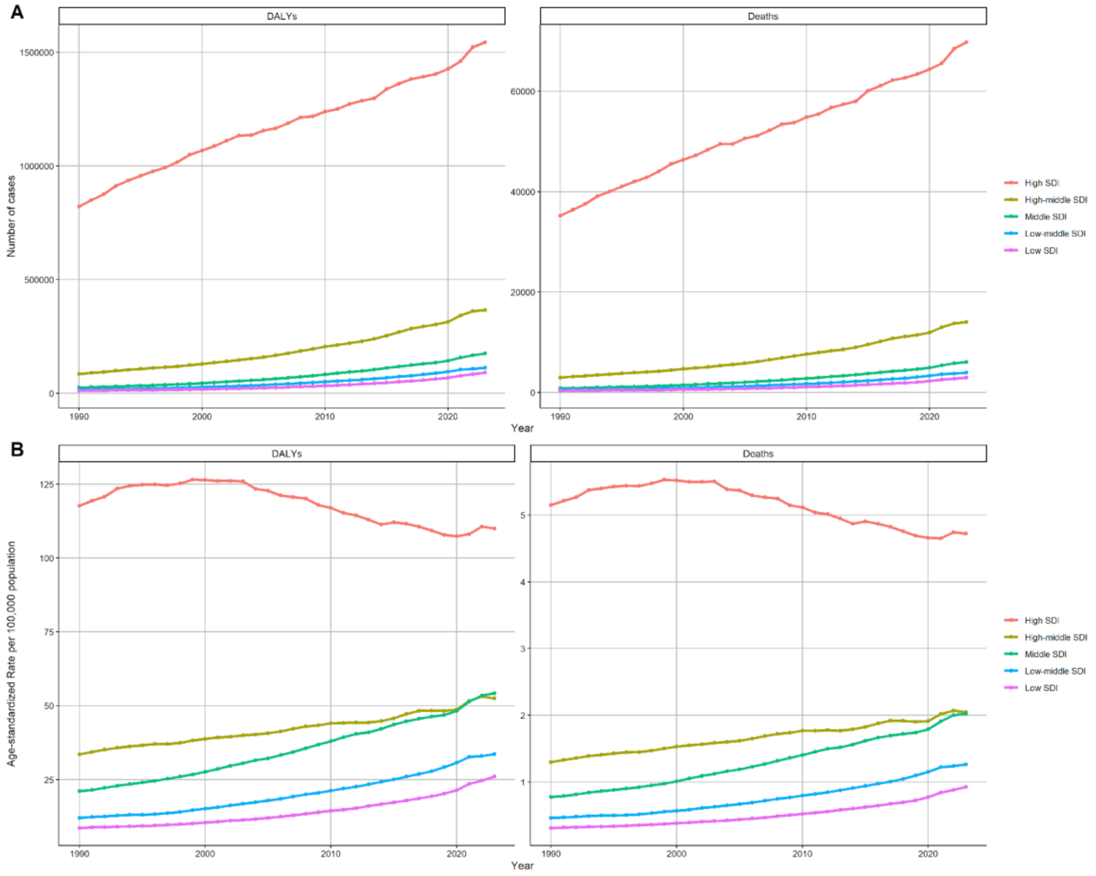

Figure S6. Results of cluster analysis based on the EAPC values of ASDRs for high BMI-related CRC deaths and DALYs over 40 years old from 1990 to 2023.

ASDRs, age-standardized rates; BMI, body mass index; CRC, colorectal cancer; DALYs, disability-adjusted life years; EAPC, estimated annual percentage change.

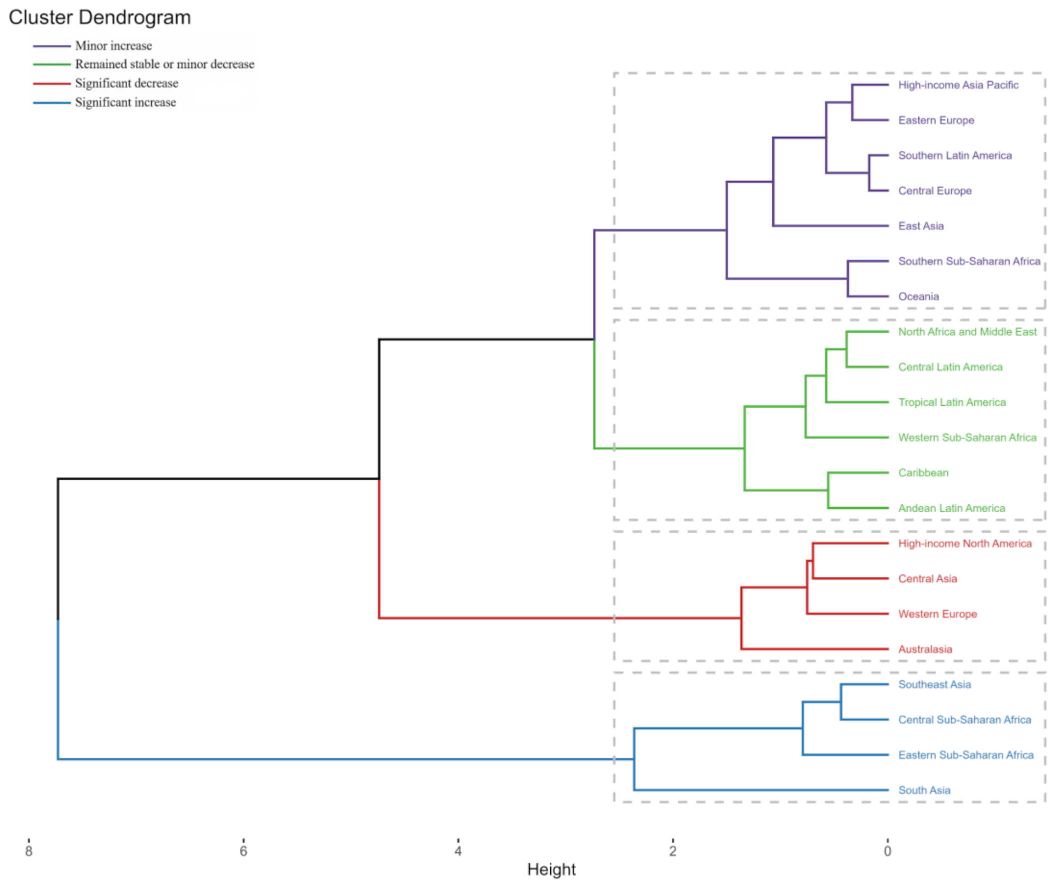

Figure S7. Temporal trends of the disease burden of CRC attribute to high BMI for adults over 40 years old from 1990 to 2023. **Panel A.** The numbers of DALYs cases and deaths cases. **Panel B.** The ASDRs of DALYs and deaths.

ASDRs, age-standardized rates; BMI, body mass index; CRC, colorectal cancer; DALYs, disability-adjusted life years.

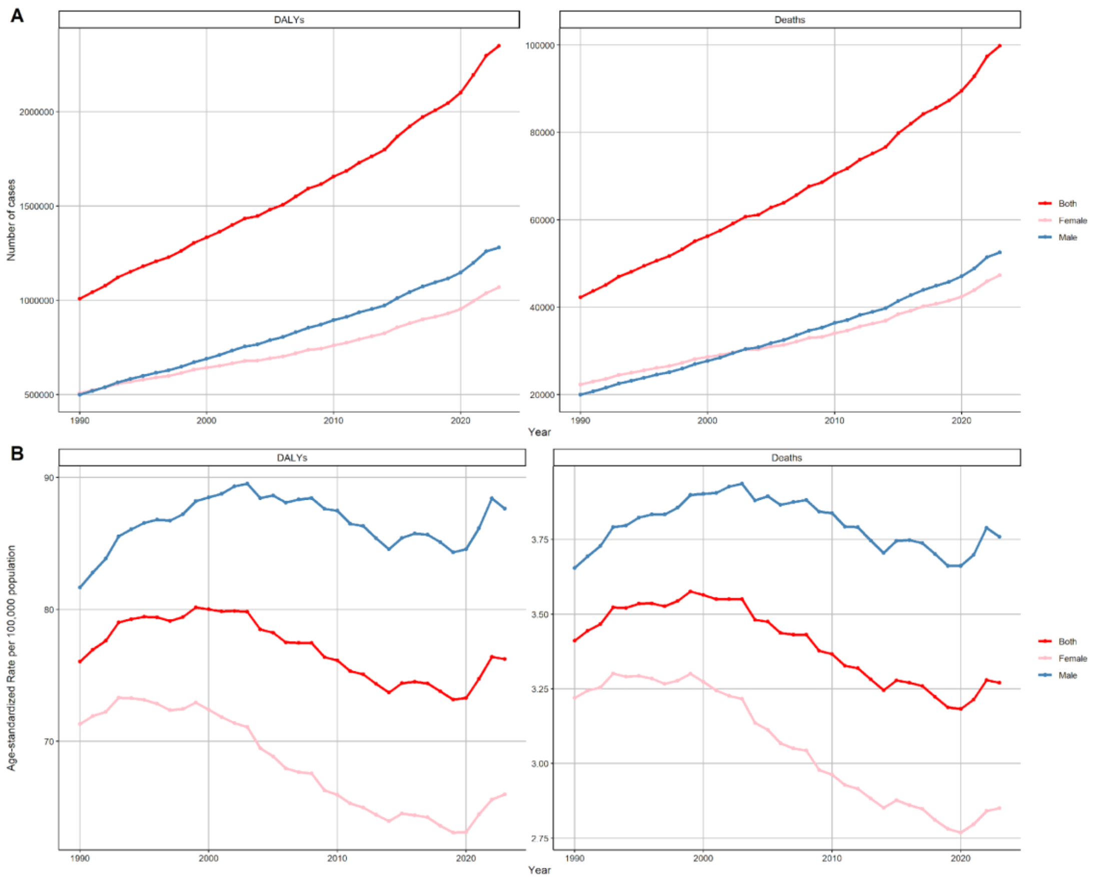

Figure S8. The disease burden of CRC in different age groups attribute to high BMI for adults over 40 years old in 2023. **Panel A.** The numbers of DALYs and deaths cases. **Panel B.** The ASDRs of DALYs and deaths.

ASDRs, age-standardized rates; BMI, body mass index; CRC, colorectal cancer; DALYs, disability-adjusted life years.

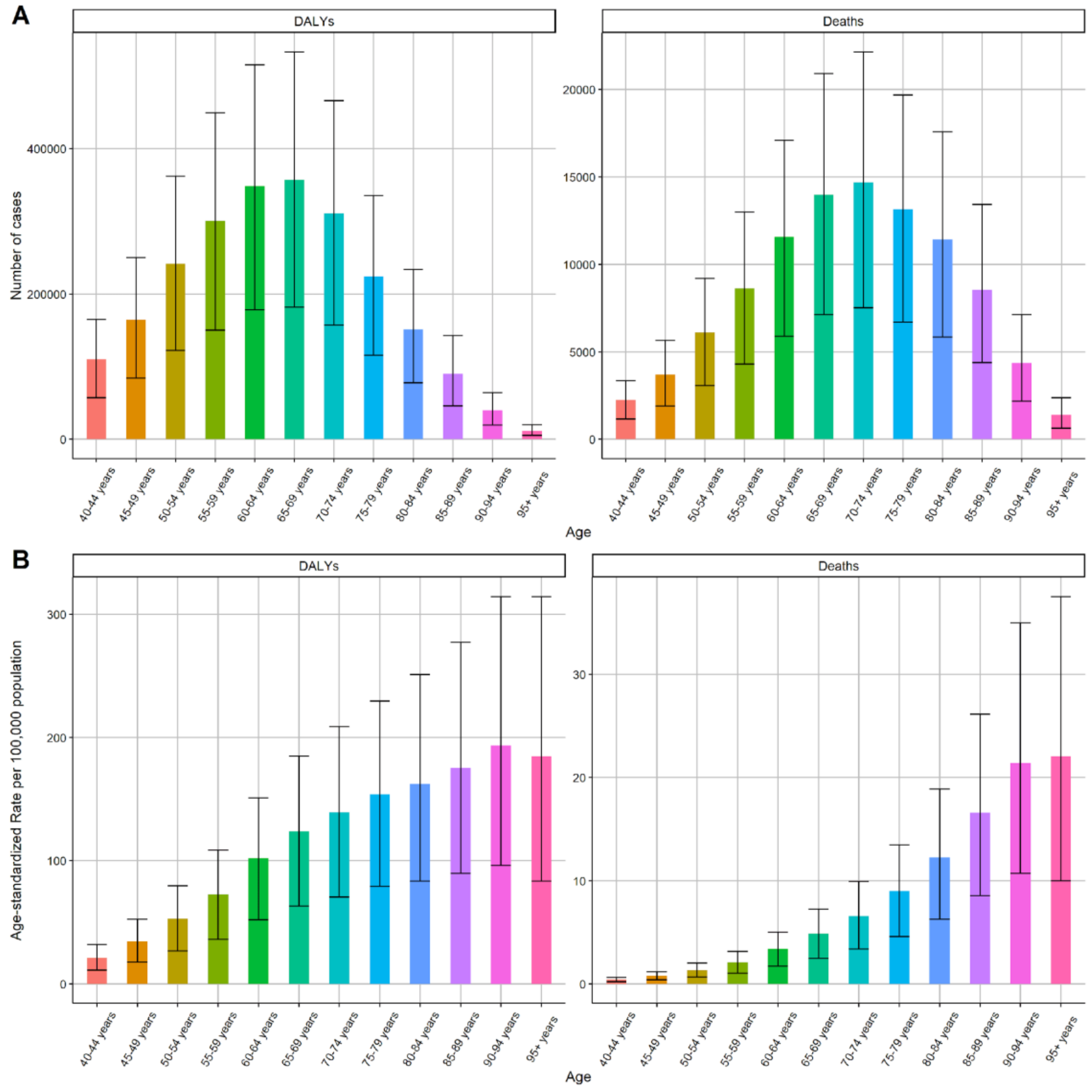

Figure S9. The age- and sex-specific disease burden of CRC attribute to high BMI for adults over 40 years old. **Panel A.** The numbers of DALYs and deaths cases. **Panel B.** The ASDRs of DALYs and deaths.

ASDRs, age-standardized rates; BMI, body mass index; CRC, colorectal cancer; DALYs, disability-adjusted life years.

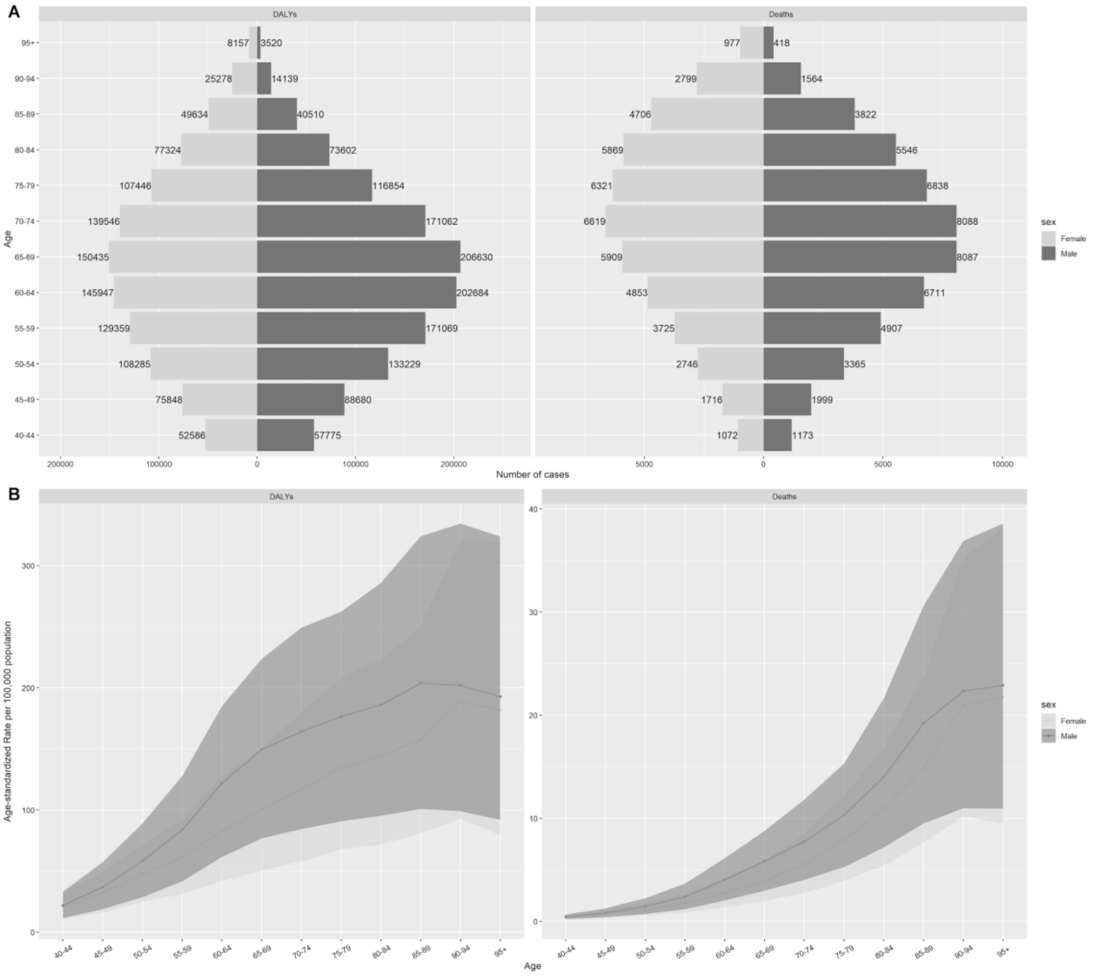

Equation S1

EAPC = $[exp(\beta) - 1] \times 100$

Detailed Methodology for the Decomposition Analysis

The method of Das Gupta (1993) was employed to decompose ΔT into the three components. This method uses a symmetric weighting scheme that averages the weights from both time points ($t1$ and $t2$), ensuring that the decomposition is exact (i.e., the components add up to the total change) and unbiased by the choice of the base year.

The total change is partitioned as follows:

$\Delta T = \Delta Pop + \Delta Age + \Delta Rate$

ΔPop is the population growth effect;

ΔAge is the population aging effect;

$\Delta Rate$ is the epidemiological effect.

$$\Delta_{Pop} = \sum_{a} \left[(N_{t2} - N_{t1}) \times \frac{(S_{a,t1} \times IR_{a,t1} + S_{a,t2} \times IR_{a,t2})}{2} \right]$$

$$\Delta_{Age} = \sum_{a} \left[(S_{a,t2} - S_{a,t1}) \times \frac{(N_{t1} \times IR_{a,t1} + N_{t2} \times IR_{a,t2})}{2} \right]$$

$$\Delta_{Rate} = \sum_{a} \left[(IR_{a,t2} - IR_{a,t1}) \times \frac{(N_{t1} \times S_{a,t1} + N_{t2} \times S_{a,t2})}{2} \right]$$

Symbol	Description	Notes
Subscripts		
t	Time (year)	e.g., $t1$ for 1990, $t2$ for 2023.
a	Age group	Denotes a specific age stratum (e.g.,40–44,45–49). $\sum_a$ indicates summation over all age groups.
Core Variables		
T_t	Total number of cases (or DALYs) in year t	This is the aggregate quantity being decomposed.
ΔT	Total change in cases (or DALYs) from $t1$ to $t2$	$\Delta T = T_{t2} - T_{t1}$
N_t	Total population size in year t	Refers to the total size of the population aged ≥40 years.
$S_{a,t}$	Proportion of the population in age group a in year t (age structure)	A proportion (between 0 and 1). Calculated as:(Population in age group a)/N_t. Satisfies $\sum_a S_{a,t}=1$.
$IR_{a,t}$	Age-specific incidence rate for age group a in year t	Typically expressed as cases per 100,000 individuals. This is a direct measure of epidemiological risk.
$P_{a,t}$	Population count in age group a in year t	$P_{a,t}=N_t \times S_{a,t}$

Table S1. The predicted results of ASDRs and DALYs by sex of the ES model.

Year	Sex	ASDR	Age-standardized DALYs rate
2024	Male	3.75998109543308	87.8344145027365
2025	Male	3.76124195023623	88.0124477414708
2026	Male	3.76237671955907	88.1726776563316
2027	Male	3.76339801194963	88.3168845797063
2028	Male	3.76431717510112	88.4466708107436
2029	Male	3.76514442193747	88.5634784186771
2030	Male	3.76588894409019	88.6686052658173
2031	Male	3.76655901402763	88.7632194282434
2032	Male	3.76716207697133	88.848372174427
2033	Male	3.76770483362065	88.9250096459922
2034	Male	3.76819331460505	88.9939833704008
2035	Male	3.76863294749101	89.0560597223686
2036	Male	3.76902861708837	89.1119284391397
2037	Male	3.76938471972599	89.1622102842336
2038	Male	3.76970521209985	89.2074639448181
2024	Female	2.86207012674315	66.4822909635585
2025	Female	2.87219794707112	66.9244419099429
2026	Female	2.8813129853663	67.3223777616888
2027	Female	2.88951651983196	67.6805200282602
2028	Female	2.89689970085105	68.0028480681744

Year	Sex	ASDR	Age-standardized DALYs rate
2029	Female	2.90354456376824	68.2929433040972
2030	Female	2.9095249403937	68.5540290164277
2031	Female	2.91490727935662	68.7890061575252
2032	Female	2.91975138442325	69.0004855845129
2033	Female	2.92411107898322	69.1908170688018
2034	Female	2.92803480408718	69.3621154046619
2035	Female	2.93156615668076	69.516283906936
2036	Female	2.93474437401497	69.6550355589826
2037	Female	2.93760476961576	69.7799120458246
2038	Female	2.94017912565648	69.8923008839824

Code for the process of analysis

Since all the authors have made great efforts to debug the correct code, readers can contact the corresponding author under reasonable circumstances. We will evaluate the actual situation and share them.

STROBE Statement—checklist of items that should be included in reports of observational studies

	Item No	Recommendation	Page No
**Title and abstract**	1	(*a*) Indicate the study's design with a commonly used term in the title or the abstract	1
		(*b*) Provide in the abstract an informative and balanced summary of what was done and what was found	2
**Introduction**			
Background/rationale	2	Explain the scientific background and rationale for the investigation being reported	3
Objectives	3	State specific objectives, including any prespecified hypotheses	3-4
**Methods**			
Study design	4	Present key elements of study design early in the paper	4-5
Setting	5	Describe the setting, locations, and relevant dates, including periods of recruitment, exposure, follow-up, and data collection	4-5
Participants	6	(*a*) *Cohort study*—Give the eligibility criteria, and the sources and methods of selection of participants. Describe methods of follow-up<br>*Case-control study*—Give the eligibility criteria, and the sources and methods of case ascertainment and control selection. Give the rationale for the choice of cases and controls<br>*Cross-sectional study*—Give the eligibility criteria, and the sources and methods of selection of participants	4
		(*b*) *Cohort study*—For matched studies, give matching criteria and number of exposed and unexposed<br>*Case-control study*—For matched studies, give matching criteria and the number of controls per case	4-5
Variables	7	Clearly define all outcomes, exposures, predictors, potential confounders, and effect modifiers. Give diagnostic criteria, if applicable	4-5
Data sources/ measurement	8*	For each variable of interest, give sources of data and details of methods of assessment (measurement). Describe comparability of assessment methods if there is more than one group	4-6
Bias	9	Describe any efforts to address potential sources of bias	6
Study size	10	Explain how the study size was arrived at	4
Quantitative variables	11	Explain how quantitative variables were handled in the analyses. If applicable, describe which groupings were chosen and why	5-6
Statistical methods	12	(*a*) Describe all statistical methods, including those used to control for confounding	4-6
		(*b*) Describe any methods used to examine subgroups and interactions	4-6
		(*c*) Explain how missing data were addressed	4,6
		(*d*) *Cohort study*—If applicable, explain how loss to follow-up was addressed<br>*Case-control study*—If applicable, explain how matching of cases and controls was addressed	4-6

Cross-sectional study—If applicable, describe analytical methods taking account of sampling strategy	
(*e*) Describe any sensitivity analyses	5

Continued on next page

**Results**			
Participants	13*	(a) Report numbers of individuals at each stage of study—eg numbers potentially eligible, examined for eligibility, confirmed eligible, included in the study, completing follow-up, and analysed	7-11
		(b) Give reasons for non-participation at each stage	7
		(c) Consider use of a flow diagram	-
Descriptive data	14*	(a) Give characteristics of study participants (eg demographic, clinical, social) and information on exposures and potential confounders	7-11
		(b) Indicate number of participants with missing data for each variable of interest	7-11
		(c) *Cohort study*—Summarise follow-up time (eg, average and total amount)	
Outcome data	15*	*Cohort study*—Report numbers of outcome events or summary measures over time	7
		Case-control study—Report numbers in each exposure category, or summary measures of exposure	-
		Cross-sectional study—Report numbers of outcome events or summary measures	-
Main results	16	(*a*) Give unadjusted estimates and, if applicable, confounder-adjusted estimates and their precision (eg, 95% confidence interval). Make clear which confounders were adjusted for and why they were included	7-11
		(*b*) Report category boundaries when continuous variables were categorized	7-11
		(*c*) If relevant, consider translating estimates of relative risk into absolute risk for a meaningful time period	-
Other analyses	17	Report other analyses done—eg analyses of subgroups and interactions, and sensitivity analyses	9-11
**Discussion**			
Key results	18	Summarise key results with reference to study objectives	12
Limitations	19	Discuss limitations of the study, taking into account sources of potential bias or imprecision. Discuss both direction and magnitude of any potential bias	15-16
Interpretation	20	Give a cautious overall interpretation of results considering objectives, limitations, multiplicity of analyses, results from similar studies, and other relevant evidence	15-16
Generalisability	21	Discuss the generalisability (external validity) of the study results	16
**Other information**			
Funding	22	Give the source of funding and the role of the funders for the present study and, if applicable, for the original study on which the present article is based	16

*Give information separately for cases and controls in case-control studies and, if applicable, for exposed and unexposed groups in cohort and cross-sectional studies.

Note: An Explanation and Elaboration article discusses each checklist item and gives methodological background and published examples of transparent reporting. The STROBE checklist is best used in conjunction with this article (freely available on the Web sites of PLoS Medicine at http://www.plosmedicine.org/, Annals of Internal Medicine at http://www.annals.org/, and Epidemiology at http://www.epidem.com/). Information on the STROBE Initiative is available at www.strobe-statement.org.
